# From growth charts to growth status: how concepts of optimal growth and tempo influence the interpretation of growth measurements

**DOI:** 10.1080/03014460.2023.2189751

**Published:** 2023-02

**Authors:** Babette S. Zemel

**Affiliations:** The Children’s Hospital of Philadelphia, University of Pennsylvania School of Medicine, Philadelphia, PA, USA

**Keywords:** Growth status, growth charts, tempo, optimal growth

## Abstract

Growth measurements are largely uninterpretable without comparison to a growth chart. Consequently, the characteristics of a growth chart become an integral component of the interpretation of growth measurements. The concepts of optimal growth and tempo are well recognised by auxologists, yet their implications for interpretation of growth measurements remain problematic. This narrative review discusses the concept of optimal growth and how it serves as a guiding principle in the development and use of growth charts. The challenges of operationalising tempo for growth assessment are also discussed. Illustrative examples highlight the importance of these two central concepts in the use and interpretation of growth measurements.

## Introduction

Over the past four and a half decades, the International Society for the Study of Human Growth and Clinical Auxology has convened an interdisciplinary group of researchers to expand our understanding of the multidimensional nature of human growth and its global variation. The Society and its Congresses have included significant contributions from clinicians, anthropologists, economists, historians, statisticians, kinesiologists, and experts in public health, nutrition, and child development. In earlier decades, secular changes in birthweight, growth and maturation, and growth differences according to social class, family size, and population ancestry were documented, propelling the recognition of the plasticity of human growth and the need to understand the mechanisms by which growth and maturation were part of the overall health and ecology of the child. Longitudinal studies were instrumental in revealing growth processes, such as the mid-childhood growth spurt (Tanner and Cameron 1980), the saltatory nature of human growth (Lampl and Johnson 1993), the relationships between growth tempo and skeletal and sexual maturation (Kozieł 2001), and methods for analysing longitudinal growth (Bock et al. 1973; Preece and Baines 1978; Jolicoeur et al. 1992; Cole et al. 2010). Further, large cohort studies have extended our insights into the intrauterine and early childhood growth and their association with adult outcomes (Victora et al. 2008) and intergenerational effects (Martorell and Zongrone 2012). The international growth reference by the WHO Multicentre Growth Study was developed (de Onis et al. 2003), intended as a standard for optimal growth of all infants and young children, and sparked intense interest in population-specific growth patterns and the consequences of a universal growth chart.

As a human biologist/auxologist situated in a tertiary paediatric care centre, I am in the position of applying these advancements in human growth to problems in research and clinical care of children. In so doing, the most salient challenges I encounter are driven by the concepts of optimal growth and tempo. This narrative review is based on my presentation at the 15th meeting of the International Society of Growth and Clinical Auxology in Vilnius, Lithuania, in 2022. Here, I focus on the use of growth charts and how the concepts of optimal growth and tempo are central yet remain problematic in growth assessment. While some of the issues are well-recognised by growth experts, they are often unknown to those who use growth measurements in interpreting research results, making clinical care decisions, or establishing public policy.

### The concept of optimal growth

There is no consensus definition of optimal growth. Upon reflection, my first thought was that optimal growth occurs when children are raised in a supportive environment that enables them to achieve their genetic potential for growth. This is similar to the rationale for the design of the WHO Multicentre Growth Study that aimed to enrol infants living in supportive environments with no known health or environmental constraints (de Onis et al. 2004). However, environmental exposures known to affect growth occur on different time scales: intrauterine, postnatal, and even transgenerational periods. The environment itself includes the natural and human-constructed environments and the familial, psychosocial, cultural, nutritional, political, and economic contexts in which the child lives. We have limited tools available with which to exclude the myriad potential adverse environmental conditions.

The plasticity of human growth, i.e. the ability to modify the growth pattern in response to the environmental context, relies on the ability of the genetic regulation of growth to respond to the environment to support survival. Genetic potential, except in broad terms, is equally difficult to define and measure, let alone gene–environment interactions. Advances in genomics have identified hundreds of single nucleotide polymorphisms (SNPs) involved in the regulation of body size. Quite remarkably, a recent genome-wide association study in a diverse sample of 5.4 million individuals, identified 12,111 independent SNPs associated with height, accounting for 40–50% of the variation in human height among those of European ancestry and 14–24% of the variation in non-European groups (Yengo et al. 2022). However, we are far from being able to predict a child’s growth potential based on their genome. Since most genome-wide association studies have been done in samples of European ancestry, we are even further away from understanding differences in genetic potential across populations and how it might be used to determine optimal growth.

A more pragmatic and appealing definition of optimal growth, defined by Cameron more than two decades ago, is “… the growth pattern that is present in conditions that result in maximum health and well-being” (Cameron 1999). Here the emphasis replaces genetic potential and “bigger-is-better” criteria with health and well-being. As the obesity epidemic has now become a global phenomenon, this is an important distinction since excess weight gain and larger body size portend both immediate and long-term adverse health consequences due to obesity. A problem with “maximum health and well-being” as the criterion to define optimal growth is the equally vague distinction of what constitutes maximum health and well-being.

Several paradigms have broached the question of what constitutes maximum health and well-being with respect to childhood growth. Seckler’s “small-but-healthy” (SBH) hypothesis proposed that populations with low height-for-age but normal weight-for-height are adapted to lower energy intake and, if no apparent functional impairments, may be considered healthy (Seckler 1984). Further, Seckler argued that “health,” not easily defined, should be based on the *absence* of disease or functional impairment. This SBH hypothesis was resoundingly rejected as a rationale that would perpetuate nutritional deficiencies with more subtle or long-term sequelae or other vulnerabilities due to limited nutritional reserves (Messer 1986). A more sophisticated model of human growth response to low energy intake derives from life history theory, which posits that energetic resources may shift from promoting growth to supporting other physiological functions such as reproduction (Ellison 2017; Wells et al. 2019) or immune function (Urlacher et al. 2018). In this paradigm, defining optimal growth according to “maximum health and well-being” is relativistic and allows for compromised growth “potential” to sustain other health domains or evolutionary success. The developmental origins of health and disease paradigm focuses on the effects of intrauterine or early life events, often associated with early growth patterns, on long-term health outcomes in adulthood (Barker 2004; Gluckman et al. 2007). Overall, the concept of optimal growth based on maximum health and well-being, functional outcomes, trade-offs for other physiological functions, or effects on adult disease requires population level longitudinal studies that test the long-term consequences of growth patterns through the lifecycle. Inherently problematic in applying the findings of such studies is the fact that the environments into which children are born and experience growth are ever changing.

Thus, the concept is fundamentally flawed as an operationalizable principle for growth assessment at the individual or population level. Nevertheless, optimal growth is an important organising concept and a driving principle in the development and selection of growth charts.

### Growth and the use of growth charts

Growth charts describe the growth attained by children of different ages. There are two important caveats here. First, growth charts are used to evaluate attained size rather than the process of growth (apart from growth velocity charts). Second, growth charts describe a statistical distribution of measurements. Cut-points to define “normal” growth are based on statistically defined thresholds, such as the 5th percentile or two standard deviations below the mean, and not necessarily on biological properties. The implicit assumption is that a growth chart defines “optimal” growth, because their application is a means to screen for “suboptimal” growth, growth failure, or growth faltering. However, this implicit assumption is only true of prescriptive charts, as explained below. Moreover, growth is a physiologic process that occurs over time and growth faltering, a disruption in physiologic processes supporting growth, can occur in a child who is large, average, or small for age (Perumal et al. 2018). Nevertheless, thresholds for normal growth are used at the population level for research or public health purposes to define the prevalence of certain growth characteristics (such as prevalence of stunting or obesity), and at the individual level, as a threshold for further testing or intervention in clinical care.

Growth charts are indispensable, however. Growth measurements, whether they are of the whole child (as in height or weight) or a tissue compartment such as lean body mass, are measures of size attained and are nearly impossible to interpret without a growth chart or reference range. Assignment of a percentile rank or *Z*-score (standard deviation score) based on the comparison of a measurement to a growth chart is an indicator of growth *status*, i.e. how a child’s growth measurement compares to peers of the same age and sex. Repeated growth measurements over time provide insight into growth as a process.

Paediatricians and public health officials are often unaware that the characteristics of a growth chart and the underlying data on which they are based become an integral component of the interpretation of growth measurements. Key considerations of growth chart development are the sampling strategy used to recruit participants, inclusion and exclusion criteria, the size and distribution of the sample (age, sex, population ancestry, geography, etc.), the accuracy and precision of the measurements, the accuracy of age determination and definition of age categories (especially important for premature infants), and the statistical approach and graphic presentation used to create the growth charts (Cole 2012). Some growth charts are “descriptive” in that they describe growth in a population with minimal or no inclusion/exclusion criteria. These are often called “reference” or “descriptive” charts since they provide a frame of reference, but not necessarily “optimal’ growth. A restricted percentile range may be used in screening for growth concerns, as in the case of the NHANES CDC 2000 growth charts for which the 5th to 95th percentile ranges are considered “normal.”

When inclusion or exclusion criteria are applied to the participants in a growth study, the resulting growth chart is “prescriptive.” The inclusion and exclusion criteria applied to the sample will identify a group of children expected to have “optimal” health and thereby define how children “should” grow. Prescriptive growth charts (sometimes referred to as a growth “standard”) use a broader range to define “normal” growth, such as ±2 SD, as in the use of the WHO Multicentre Growth Study charts. Other charts may be a blend of prescriptive and descriptive samples. For example, very low birthweight infants were excluded from the CDC 2000 infant growth charts. For the CDC 2000 BMI charts, BMI data from 1963 to 1994 were used for children less than 6 years of age, but for children above the age of 6 years data from the from the NHANES III survey (1988–1994) were excluded because of the growing prevalence of obesity in children and adolescents recognised at the time the survey was completed (Kuczmarski et al. 2002).

These distinctions of descriptive versus prescriptive growth charts are extremely important for interpreting growth status of an individual or prevalence of faltering in a population. The prescriptive charts from the WHO Multicentre Growth Study were intended to provide an international standard of optimal human infant growth based on healthy, breastfed babies from different continents. The consensus that population differences in linear growth during infancy and early childhood are minimal among well-fed children was further rationale for the development and promotion of the WHO Multicentre Study as an international growth standard (de Onis et al. 2003). However, numerous studies have evaluated the applicability of these international growth charts in different populations, as summarised beautifully by Thompson (2021). Given known population variation in body size, use of an international standard can result in overdiagnosis of children with “poor” growth in populations with smaller body size, leading to unnecessary testing and distress for parents. Similarly, in populations with larger body size, there are concerns of potential underdiagnosis of “poor” growth in populations, leading to missed evaluations of health concerns.

Far less attention has been given to growth assessment in school age children and adolescents. The WHO growth references for children aged 5–20 are based on a reconstruction of the US 1977 National Centre for Health Statistics growth charts. The reconstruction used the “LMS” method (Cole and Green 1992) to construct reference percentiles with a smooth transition from the WHO Multicentre growth charts for children less than 5 years of age (de Onis et al. 2007). The data used were collected prior to the rapid rise in obesity in the United States and are presumed to be representative of the US population at the time of data collection. The fact that the source sample is not geographically diverse is potentially a major limitation in its use as an international reference. In addition, variability in growth tempo is not incorporated into these charts and tempo can have a profound effect on growth “status” in the years surrounding pubertal development and peak growth velocity.

By questioning whether it is possible to accept a universal standard for optimal growth, the considerations given to the development of growth charts comes into sharper focus. The following examples illustrate the implications of the issues discussed above and their impact on the interpretation of growth measurements.

#### Example 1: Growth assessment in preterm infants

Infants born preterm face numerous health-related challenges and growth assessment is critical for informing nutritional care and risk of health complications. Meeting nutritional needs for growth in preterm infants is challenging, whereas the delivery and absorption of nutrients *in utero* is highly efficient. Therefore, the growth at the time of birth of infants at different gestational ages is considered close to “optimal.” Indeed, relative weight gain in preterm infants postnatally, as assessed by BMI, is substantially different from the distribution of BMI values of infants of similar gestational age at birth (Williamson et al. 2018).

In 2010, we published intrauterine growth charts based on data collected at birth for 257,855 singleton births born at 22–42 weeks gestation (Olsen et al. 2010, 2015). Data were collected from 248 hospitals located in 33 states in the United States between 1998 and 2006. The new sex-specific curves for weight, length, head circumference, and BMI were an advancement over prior curves used in North America because of the diversity and size of the sample and the fact that all three measures were collected on the same set of infants. Infants were excluded if they were multiple births, had congenital anomalies, or died before discharge. The racial and ethnic diversity of the cohort was comparable to the US population at the time of data collection, categorised as 50.6% white, 15.7% black, 24.4% Hispanic, and 9.3% other. For preterm infants, classifications for small-for-gestational age (SGA, < 10th percentile for weight-for-gestational age) and large-for-gestational age (> 90th percentile for weight-for-gestational age) are widely used markers of increased risk of mortality, neurodevelopmental outcomes, and metabolic abnormalities that inform treatment decisions and provide anticipatory guidance for families.

Several years later, another set of growth charts was published for infants of 22–29 weeks gestational age based on a sample of 93,951 singleton infants born between 2006 and 2014 from 852 centres in the United States and Puerto Rico (Boghossian et al. 2016). The sample was described as 50.3% White, 34.3% Black, 3.9% Asian, and 12.5% Hispanic. The distribution of reference percentiles was lower than values published by Olsen et al. (2010), resulting in significant differences in the categorisation of small-for-gestational age using these two growth charts. For example, for infants 22–25 weeks gestational age, 13% of infants in their validation cohort were classified as SGA using the charts of Olsen et al. (2010), whereas 9.1% of infants were classified as SGA using their new charts (Boghossian et al. 2016). The actual difference in values at the 10th percentile was small but the effect on the prevalence of SGA differed by 4%. There are several subtle differences in methods between the two studies that can account for these differences. First, Olsen et al. used completed weeks of gestation as the age indicator, whereas Boghossian et al. used number of days. This could result in a leftward shift in the distributions of weight-for-age (Clark and Olsen 2016). In addition, the composition of the cohorts was different in terms of population ancestry. Boghossian et al. showed that the weight-for-age distribution for the infants whose mothers were black was lower than that for infants whose mothers were white (Boghossian et al. 2016). This racial disparity has been reported previously. The difference in the racial and ethnic composition of the two cohorts (15.7% vs 34.3% black) could further add to the difference in the curves and resulting prevalence of SGA.

Another source of reference data for preterm infants is the INTERGROWTH-21st project, that enrolled healthy pregnant women from eight geographical diverse urban populations and assessed foetal growth by ultrasound and newborn size (Villar et al. 2014, 2016). The study was intended to parallel the World Health Organisation Multicentre Growth Reference Study (de Onis et al. 2004). The number of preterm infants in the study was much smaller than the previously described studies (408 infants 24–32 weeks gestational age, and 406 infants 33–36 weeks gestational age). Notable for these charts was that the 90th percentile for the INTERGROWTH-21st charts was lower than the Olsen charts. When the prevalence of SGA and LGA based on the Olsen et al. (2010) and INTERGROWTH-21st charts (Villar et al. 2016) were compared using data on 192,888 infants from US neonatal intensive care units (Ferguson et al. 2020), there were substantial differences, especially at older ages.

These examples of growth charts for preterm infants illustrate how both blatant (e.g. sample size, population ancestry) and nuanced (age determination) characteristics of study design impact reference percentiles and the identification of children who are large or small for gestational age. The use of cut points, such as the 10th and 90th percentiles to define diagnostic categories, is somewhat arbitrary, but these cut points are known to be associated with health outcomes. Realistically, there is a continuum of health risk associated with smaller and larger body size at birth and practitioners use growth status along with other indicators in clinical care decisions.

#### Example 2: Growth charts for children with genetic conditions

Altered growth, body composition, body proportions, and pubertal timing are common among children with a wide variety of health conditions caused by genetic disorders. Families and care providers have a strong interest in knowing how a child is growing in comparison to others with the same health condition. Growth charts for a wide range of groups with genetic syndromes are available (Brook and Dattani 2012; Hall et al. 2012). The development and use of disease specific growth charts involves special considerations. First, are growth patterns (usually short stature) a result of the genetic condition itself or are they secondary to other disease-related processes that alter dietary intake or nutrient requirements? Second, many genetic conditions of concern are relatively rare. Are there enough children of each age and sex group to capture the variability in growth outcomes? Third, have there been advancements in medical care that may result in improved growth, such that the context in which younger children now live is different from that of 10 or 20 years ago? Fourth, are there health disparities, including access to care and support resources, that affect growth and health outcomes and, if so, are these captured in the sampling strategy for development of growth charts? The seriousness of these concerns relates back to the definition of optimal growth, the distinction between prescriptive and descriptive growth charts, and how growth status is interpreted. If growth patterns are a primary result of a genetic condition, then growth charts for that group of children may reflect their genetic potential. However, if growth alterations are secondary to disease-related processes, then descriptive charts have the potential to institutionalise poor growth.

Growth charts for children with Down syndrome (DS) illustrate some of these issues. DS (Trisomy 21) is a genetic condition estimated to occur in one in 732 live births in the United States (Canfield et al. 2006). People with DS typically have short stature, microcephaly, a tendency to overweight status, and a spectrum of health complications and physical and cognitive disabilities. Infants with DS, on average, have lower birth weights than typical children (Anneren et al. 1993). Delayed oromotor development and poor muscle tone have the potential to limit nutrient intake and contribute to growth faltering in early life (Bull and Committee on Genetics 2011). Life expectancy of people with Down syndrome in the United States increased from 35 years in 1982 (Thase 1982) to 53 years in 2007, in part due to advances in care, such as correction of cardiac defects and reduced institutionalisation (Presson et al. 2013). During childhood and adolescence and into adulthood, the prevalence of obesity increases indicating that energy intake is more than adequate to meet needs for physical activity and growth.

Growth charts for infants and youth with Down syndrome in the United States were published in the 1980s based on data compiled from prior research projects and medical chart review of children with DS conducted at multiple centres (Cronk 1978; Cronk et al. 1988). The population ancestry and socioeconomic characteristics of the sample were not described, and the study does not specify the dates that the data were collected or the range of birthdates of children included in the sample. The manuscript was accepted for publication in 1986, so one might estimate that the children studied would have likely been born between 1967 and 1985.

The community of families and care providers of children with DS became increasingly concerned about the relevance of these growth charts as more recent growth curves for children with DS from other countries were being published (Cremers et al. 1996; Myrelid et al. 2002; Styles et al. 2002; Kimura et al. 2003; Meguid et al. 2004). Of particular interest were growth charts from the United Kingdom and the Republic of Ireland based on medical chart review data for over 1,000 children and 5,000 observations (Styles et al. 2002), with 94% of the sample of European ancestry. The exclusion criteria applied to the sample used to develop these curves were: children who were deceased, had a coexistent major pathology, or history of cardiac surgery. Measurements of preterm infants were excluded if they were obtained during the first 2 years of life, but they were included at older ages. When compared with the US growth charts from the 1980s, the growth in height between 3 and 12 years of age were similar but, at older ages, the distribution of heights for males was lower for those in the United States compared to those in the United Kingdom.

Concerns about the inadequacy of the US charts led to a cooperative project with the Centres for Disease Control and Prevention to develop new growth charts for children with DS based on prospectively collected, research quality measurements. The Down Syndrome Growing Up Study (DSGS) (Zemel et al. 2015) enrolled 637 children (51% male, 9% Hispanic, 11% non-Hispanic black/African American, and 73% non-Hispanic white/European ancestry) and acquired 1,537 measurements. Exclusion criteria included other major genetic disorders associated with altered growth (e.g. sickle cell disease), very low birth weight (< 1,500 g), or if they were not in a usual state of health (e.g. cancer therapy) at the time of measurement.

**Figure 1.**
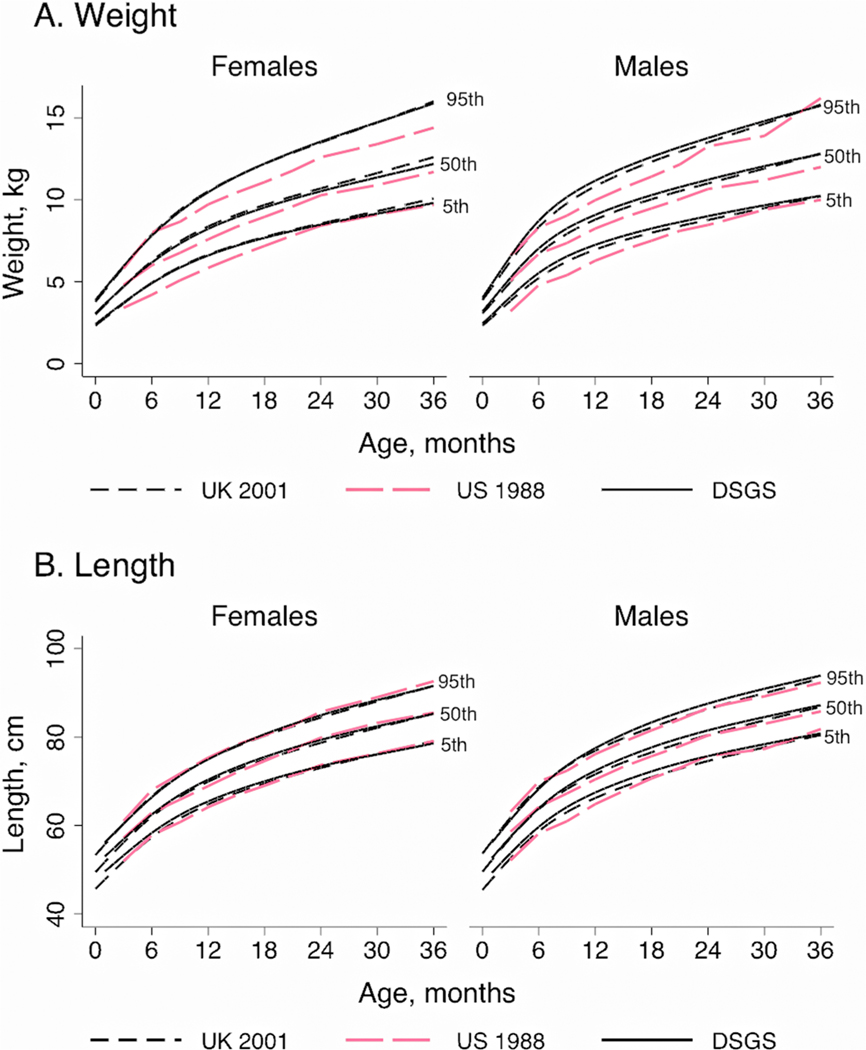
Comparison of growth charts for infants and children with Down syndrome, 0–36 months of age. The UK 2002 charts (Styles et al. 2002) were compared to charts from the US 1988 (Cronk et al. 1988) and the Down Syndrome Growing Up Study (DSGS) (Zemel et al. 2015). Shown are the 5th, 50th, and 95th percentiles for weight (A) and length/height (B) for males and females.

For children less than 36 months of age, the new (DSGS) US charts (Zemel et al. 2015) demonstrated substantial improvements in growth in weight for boys and girls, and for length in boys compared to the 1988 US curves (Cronk et al. 1988). The new US charts were very similar to the UK charts (Styles et al. 2002) for this age range (Figure 1).

**Figure 2.**
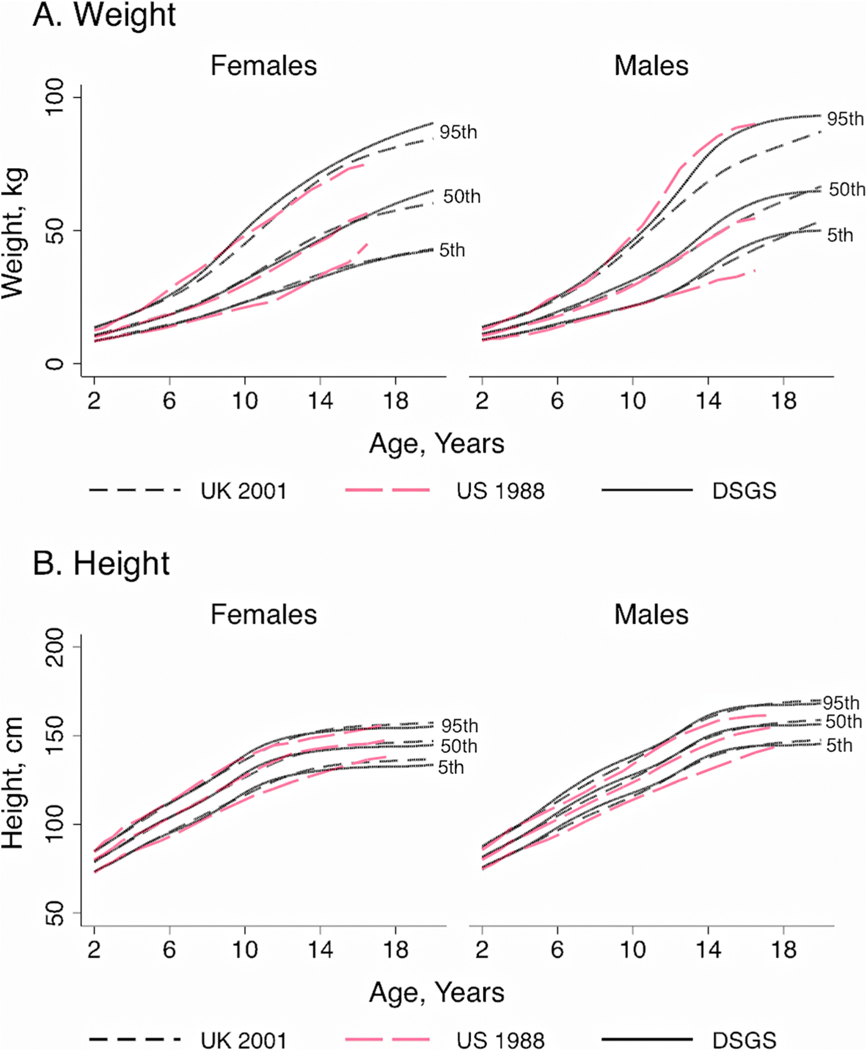
Comparison of growth charts for children and adolescents with Down syndrome, 2–20years of age. The UK 2002 charts (Styles et al. 2002) were compared to charts from the US 1988 (Cronk et al. 1988) and the Down Syndrome Growing Up Study (DSGS) (Zemel et al. 2015). Shown are the 5th, 50th, and 95th percentiles for weight (A) height (B) for males and females.

For older children (2–20 years), the boys from the newer US (DSGS) study were taller than the 1988 US curves at most ages, whereas all three height curves for girls were somewhat similar (Figure 2). For weight, the newer US 95th percentile of older girls (> 8 years) and the 5th and 50th percentiles for older boys (≥ 12 years) were greater, yet the 95th percentile for boys was lower than the corresponding percentiles from the older 1988 US growth charts. Overall, the new US growth charts for length and height were similar to those from the United Kingdom.

The DSGS also published BMI charts for children 2–20 years of age (Zemel et al. 2015). In part, the rationale for DS-specific BMI charts was based on the known differences in body proportions in people with DS, specifically shorter limb lengths, compared to the general population. This may affect BMI distributions. Nevertheless, the publication of DS-specific BMI charts evoked controversy because of the high rate of obesity among children with DS. This concern mirrors concerns in the general population regarding the use of contemporary data to create BMI charts for the general population (as discussed above). As noted in the news release regarding the new DS growth charts by the American Academy of Paediatrics, “The growth curves … represent current trends but not necessarily optimal growth.” Dr. Marilyn Bull, author of the Academy’s 2011 clinical report on Down syndrome, said “… children with Down syndrome tend to have low metabolic rates, and some have poor diets. Until optimal BMI guidelines for individuals with Down syndrome are established, clinicians should use the BMI guidelines of the CDC charts” (Jenco 2015).

Indeed, the DSGS had minimal exclusions and was not designed to be a prescriptive chart. Therefore, use of the DS BMI charts would provide information about how an individual child or group of children with DS compared to a contemporary group of children with DS, with no regard to the health risks associated with excess adiposity.

To address this knowledge gap, we compared adiposity assessed by dual energy x-ray absorptiometry in children with DS to the CDC 2000 BMI charts (Ogden et al. 2002) and the DSGS growth charts (Zemel et al. 2015) in a cohort of 121 youth with DS, ages 10–20 years (Hatch-Stein et al. 2016). The sensitivity and specificity of the 85th percentile for the two BMI charts were compared using fat mass index ≥ 80th percentile as a criterion. The excellent sensitivity of the CDC 85th percentile to identify children with high FMI (100% vs 62% for the DS 85th percentile for BMI), provided evidence for supporting the use of the CDC 2000 BMI charts to evaluate relative weight in older children and adolescents with DS. Further work is needed to determine the best approach to evaluate excess adiposity in children less than 10 years of age.

In sum, the history of growth charts for children with DS illustrates the challenges in developing disease-specific growth charts for a group with a genetic syndrome that alters growth patterns. DS is a relatively common genetic disorder, yet acquiring a sufficient sample size to generate growth charts requires a broad reach across multiple centres and geographic regions. The improvements in care, especially treatment of cardiac malformations and therapies to improve nutritional status in infants, are evident in the differences between the US growth charts in 1988 versus 2014 for infants and young children. The modest changes in linear growth of older children during this time interval suggests that current US and UK growth charts, which are remarkably similar, may be representative of the growth potential of children with DS. Yet DS-specific charts for BMI are far from optimal as their use would “institutionalise” the acceptance of excess adiposity in children with DS. Indeed, our studies demonstrated that BMI ≥ CDC 85th percentile was associated with increased risk of prediabetes and dyslipidemia (Magge et al. 2019) in youth with DS, providing further justification for the use of the CDC 2000 growth charts for screening for excess adiposity in DS. The guiding principles of genetic potential and optimal health support the use of this blend of growth charts for children with DS.

### Growth and the concept of tempo

The concept of tempo (Tanner 1962) is central to understanding variation in growth. Individuals vary in their timing of maturational stages (i.e. pubertal or skeletal maturation) or events (i.e. age at menarche or peak height velocity), and these events are closely tied to the size and composition of the body because of their common hormonal drivers. Children with a more rapid tempo and earlier maturation are transiently taller relative to their peers and have body composition profiles that are more similar to older children, so knowledge of tempo is valuable for growth and body composition assessment.

Tempo is not easily assessed, except in retrospect when the entire growth record can be reviewed to determine the timing of peak height velocity, the timing of events such as menarche once they have already occurred, or progress through puberty. Relative skeletal maturation, the comparison of skeletal age to chronological age to determine advanced or delayed skeletal maturation is an indicator of tempo, but once skeletal maturity is attained it can no longer be used. Skeletal age is used for prediction of adult height in the clinical setting (Bayley and Pinneau 1952; Tanner et al. 1983, 2001), and the percentage of adult height attained based on different stages of skeletal maturity is fairly uniform (Sanders et al. 2017). However, to my knowledge, there are no validated guidelines on how bone age should be used in determining growth status. Common practice is to substitute bone age for chronological age in using a growth chart, but it is unknown whether the distribution of growth outcomes for children of a given bone age are the same as the distribution for children of a comparable chronological age. For example, it is unlikely that the distribution of heights for 12 year old girls is the same as the distribution of heights for girls with a bone age of 12 years. Alternatively, Tanner and Davies (1985) published growth charts for height and height velocity for early, average and later maturing children, so relative skeletal maturation could be used to assign maturation status. However, the categorisation of early or late maturation for these growth charts was based, not on relative skeletal maturation, but on timing of peak height velocity (beyond ±2 SD) in the Harpenden Growth Study (Tanner et al. 1976) and superimposed on the NCHS growth charts (Hamill et al. 1977). Presently, there are no well-developed methods for incorporating bone age into growth assessment. Moreover, skeletal age is frequently not available in the research or public health setting.

**Figure 3.**
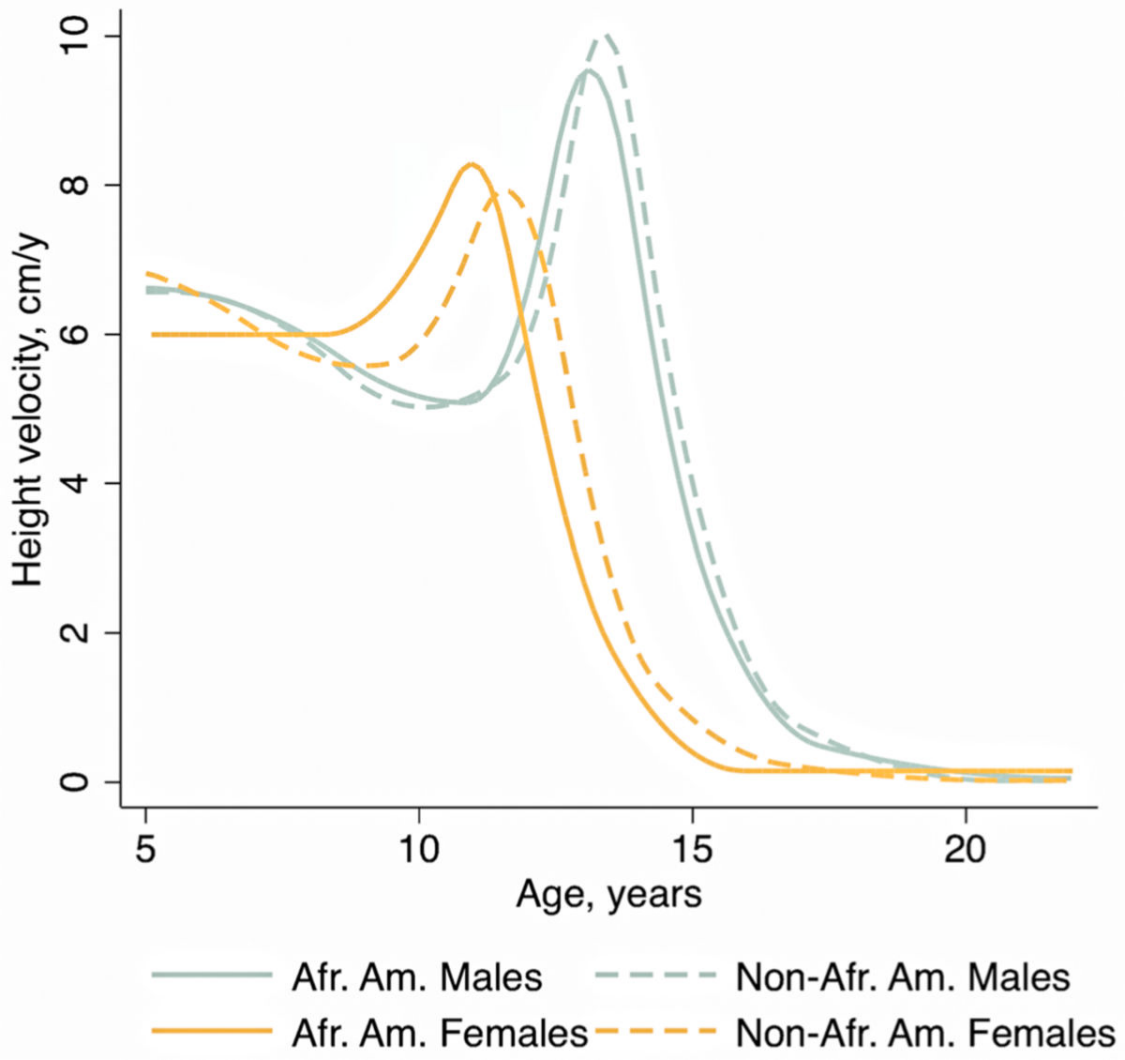
Example of siTAR modelling applied to height growth of African American (AA) versus non-African American (non-AA) male and female participants from the Bone mineral Density in Childhood study (from mcCormack et al. 2017). Height velocity curves illustrate the difference in timing of peak height velocity (tempo) between AA and non-AA girls, and between males and females.

In the research setting, tempo can be quantified using SITAR (Simultaneous Translation and Rotation), a highly innovative statistical modelling approach, developed by Cole et al. (2010). This modelling approach describes longitudinal growth in a sample of children in terms of “size,” “tempo,” and “velocity.” It can be used to compare different samples or, within a sample, how individuals deviate from the population mean curve. An example of SITAR applied to growth of African American versus non-African American males and females from the Bone Mineral Density in Childhood Study is shown in Figure 3 (McCormack et al. 2017). This study illustrates the known differences in age at peak height velocity (PHV) for males versus females, and the earlier age at PHV among African American compared to non-African American youth for females: mean 11.0 years (95% CI = 10.8–11.1) versus 11.6 years (95% CI = 11.5–11.6, *p* < 0.001) and males: mean 13.1 years (95% CI = 13.0–13.2) versus 13.4 years (95% CI = 13.3–13.4, *p* < 0.001).

Stage of pubertal maturation can be useful for assessing tempo but has several limitations. Puberty stage assessment requires skill and a private setting to conduct the evaluation. Both parents and children are reluctant regarding this type of examination and there is potential to cause emotional distress. It is rarely available in survey data, school or research settings, or primary care. Timing of pubertal onset and age at entry into pubertal stages have been described in the United States and differences between racial and ethnicity groups reported (Sun et al. 2002; Wu et al. 2002). Except for children whose puberty stage for age is outside the wide range of normal variation, there is no way to quantify tempo, i.e. are they earlier or later maturing than their peers and to what degree?

**Figure 4.**
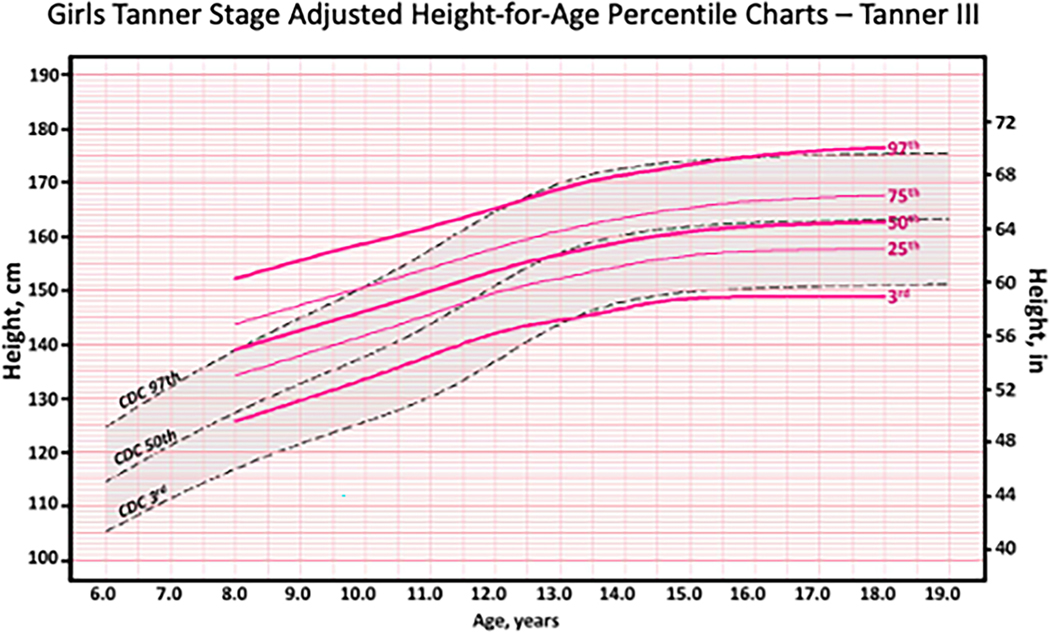
Example of Tanner stage adjusted height-for-age percentile chart for girls in Tanner stage III. From Miller et al. (2020, appendix Figure 10).

Recently, an approach incorporating Tanner stage into height assessment was developed with data from the US National Health and Nutrition Examination Survey (Miller et al. 2020). The sample was comprised of 13,358 children, 9–18 years old, from multiple US surveys. Reference ranges for height-for-age for each Tanner stage (breast stage for girls and genital stage for boys) were created. The resulting curves are illustrated in Figure 4 showing the height curves for girls in Tanner Stage 3 compared to the CDC 2000 height curves.

The Tanner stage adjusted height curves were used to examine the prevalence of extremes of stature (very tall or very short) in different race-ethnicity groups in the United States (Addo et al. 2018). Since this approach accounted for differences in pubertal timing between groups, the estimated prevalences of shortness and tallness were greatly reduced. For example, the percentage of males categorised as short (height for age *Z*-score < −1) based on chronological age alone was 8.6%, 16.5% and 6.9% for non-Hispanic White, Mexican American and Non-Hispanic black groups, respectively but, using Tanner Stage adjusted scores, the prevalences were 15.3%, 12.6% and 15.1%, respectively. Adjusting for differences in pubertal timing also reduced the prevalence of overweight and obesity, especially in Mexican Americans and non-Hispanic Black youth (Bomberg et al. 2021).

This approach holds promise for accounting for the effects of tempo on growth assessment when puberty stage information is available. Further study is needed to determine the relationship between tempo-adjusted growth status and health outcomes to fully appreciate the relationship between tempo and optimal growth.

#### Summary

Growth measurements need to be compared to growth charts to determine growth “status,” i.e. a percentile rank or standard deviation *Z*-score quantifying a child’s growth relative to peers. The relative nature of growth status is often taken for granted and carries the assumption that the growth chart used provides an absolute basis of comparison. The nuances of how growth charts are developed thereby assumes great importance. If a growth chart captures “optimal” growth, then the basis of comparison is strong. However, the concept of optimal growth is abstract since factors such as genetic potential, health and well-being and environmental stressors cannot be fully measured. Nevertheless, the concept of optimal growth provides guiding principles for evaluating growth charts with questions such as: is population variation in genetic growth potential adequately represented in the source sample? What measures of health or child environment were used to restrict the source sample? Is there an adequate number of individuals in each age/sex cell to characterise the range of variation?

Examples of growth charts for preterm infants and children with Down syndrome provide excellent examples of the impact of study design on the use and interpretation of growth measurements. For preterm infants, weight and length at birth were used in chart development since the intrauterine environment is the most optimal to support growth. Differences in the racial/ethnicity distribution in the source samples as well as sample size provide good explanations for observed differences in prevalence of SGA and LGA according to different growth charts.

Disease-specific growth charts are widely considered valuable for children with genetic syndromes that affect growth. The contemporary growth charts for children with DS in the United States and United Kingdom show strong similarities and both differ from older charts for infants and young children. The supportive care and environment of contemporary children with DS may be reflected in their growth status, and these newer charts represent more “optimal” growth for this group of children. However, the increasing prevalence of excess adiposity with age in children with DS provides strong rationale for rejecting the use of DS-specific charts for weight or BMI in older children. Indeed, using the CDC 2000 growth charts to identify overweight more accurately captured excess adiposity and cardiovascular disease risk in youth with DS. Disease-specific charts are not always optimal for health outcomes.

Methods for incorporating tempo into growth assessment are severely limited. Tempo itself is difficult to measure, except in retrospect after events such as peak height velocity have already occurred. Pubertal timing has a strong genetic component (Cousminer et al. 2016), yet the secular trend and environmental correlates with age at menarche are well documented (Eveleth and Tanner 1991). Growth status of peripubertal children is strongly influenced by pubertal timing and easily accessible methods for evaluating growth independent of tempo are needed. The development of Tanner stage adjusted growth charts are a major innovation in the field but need to be vetted further to determine how Tanner stage adjusted growth status relates to long-term health and well-being.
